# Research progress in the management of vascular disease with cannabidiol: a review

**DOI:** 10.1186/s13019-023-02476-y

**Published:** 2024-01-03

**Authors:** Yilong Guo, Ren Wei, Jianqing Deng, Wei Guo

**Affiliations:** 1 Medical School of Chinese PLA, Beijing, 100037 China; 2 Department of Vascular and Endovascular Surgery, The First Medical Centre of PLA General Hospital, 28#, Fuxing Road, Beijing, 100037 China; 3 Senior Department of Cardiology, The Six Medical Centre of PLA General Hospital, Beijing, 100037 China

**Keywords:** Vascular disease, Drug therapy, Cannabidiol, Molecular target, Research progress

## Abstract

The morbidity and mortality rates associated with vascular disease (VD) have been gradually increasing. Currently, the most common treatment for VD is surgery, with the progress in drug therapy remaining slow. Cannabidiol (CBD) is a natural extract of Cannabis sativa L. with sedative, analgesic, and nonaddictive properties. CBD binds to 56 cardiovascular-related receptors and exerts extensive regulatory effects on the cardiovascular system, making it a potential pharmacological agent for the management of VD. However, most CBD studies have focused on neurological and cardiac diseases, and research on the management of VD with CBD is still rare. In this review, we summarize the currently available data on CBD in the management of VD, addressing four aspects: the major molecular targets of CBD in VD management, pharmacokinetic properties, therapeutic effects of CBD on common VDs, and side effects. The findings indicate that CBD has anti-anxiety, anti-oxidation, and anti-inflammatory properties and can inhibit abnormal proliferation and apoptosis of vascular smooth muscle and endothelial cells; these effects suggest CBD as a therapeutic agent for atherosclerosis, stress-induced hypertension, diabetes-related vasculopathy, ischemia-reperfusion injury, and vascular damage caused by smoking and alcohol abuse. This study provides a theoretical basis for further research on CBD in the management of VD.

## Background

Vascular disease (VD) refers to structural damage and dysfunction of the vascular tissue from various causes such as smoking, high cholesterol, and inflammation. Common vascular diseases include atherosclerosis, arterial occlusion, and vasculitis. Recently, the morbidity of hypertension, hyperlipidemia, and diabetes mellitus has been rising, leading to a gradual increase in the morbidity of VD [1, 2]. Currently, the most common treatment for VD is surgery, with drug therapy progressing slowly. However, drug therapy is important in particular patients for whom surgery is not feasible (patients with surgical contraindications or who cannot tolerate surgery due to poor general conditions) [3, 4]. Therefore, there is an urgent need to develop new pharmacological agents for VD treatment.

Marijuana has been used for entertainment, medical treatment, and mental control for thousands of years [5]. However, it was not until the middle of the 20th century that its major constituents, delta-9-tetrahydrocannabinol (D9-THC) and cannabidiol (CBD), were first purified [6]. Owing to its strong psychostimulant properties and toxic side effects, D9-THC has been classified as an illicit drug. However, CBD is a 21-carbon terpene phenolic compound with no obvious addictive or severe toxic side effects [7, 8]. Studies showed that CBD acts on more than 65 receptors, and its potential pharmacological actions include sedation, analgesia, anti-anxiety, anti-oxidation, and inhibition of inflammation and abnormal proliferation and apoptosis of vascular smooth muscle cells (VSMCs) and endothelial cells (ECs) [8–10]. As the potential pharmacological actions of CBD described above are closely related to the pathophysiology of VD, we speculate that CBD could be useful in the management of VD.

This review summarizes the pharmacological actions of CBD and explores the feasibility of its use in the management of VD. Given that clinical studies on the use of CBD in the management of VD are limited, most of the results included in this review were obtained from studies using animal disease models.

## Article search methods

The articles reviewed during writing this manuscript were extracted from PubMed using the following search strategy: “((((((((coronary artery disease [Text Word]) OR (Hypertension [Text Word])) OR (diabetes [Text Word])) OR (Ischemia reperfusion injury [Text Word])) OR (smoking [Text Word])) OR (alcoholism [Text Word]))) OR (vascular disease [Text Word])) AND ((CBD [Title]) OR (cannabidiol [Title])) AND (English [Filter]).” Overall, 157 papers were identified; after screening the abstracts, 70 papers were excluded (48 were excluded because the studies were not mainly focused on the therapeutic effects of CBD in vascular disease; 22 were excluded because they included the use of CBD derivatives). Finally, 87 articles were included for this study.

### Major molecular targets of CBD related to VD management

Previous studies have shown that CBD has 56 cardiovascular-related targets. Among them, the major targets related to the management of VD are cannabinoid receptor 1 (CB1), cannabinoid receptor 2 (CB2), transient potential vanilloid channel 1 (TRPV1), 5-hydroxytryptamine 1 A receptor (5-HT1A), peroxisome proliferator-activated receptor γ (PPARγ), and cyclooxygenase isoenzymes (including COX-1 and COX-2) [11]. CB1 and CB2 are important components of the endocannabinoid system (ECS); TRPV1 has neuroprotective, anticonvulsant, antipsychotic, and immune regulatory effects; and 5-HT1A alleviates mental illnesses and mediates cell survival [12–14]. The major molecular targets of CBD related to the management of VD are listed in Table 1.

**Table 1:** Major molecular targets of CBD related to the management of VD

Molecular targets	Classification	Regulatory function of CBD	References
ECS:			
CB1	Receptor	CB1 is located mainly in the central nervous system and its activation promotes oxidative stress and inflammation; CBD is a negative allosteric modulator of CB1.	[21–25]
CB2	Receptor	CB2 is located mainly in the immune system and its activation inhibits oxidative stress and inflammation; CBD is an inverse agonist of CB2.	[21–25]
AEA	Endogenous cannabinoid	CBD administration has been shown to increase the concentration of AEA.	[21, 29]
2-AG	Endogenous cannabinoid	CBD administration has been shown to increase the concentration of 2-AG.	[23, 29]
FAAH	Metabolic enzyme	CBD inhibits the major endogenous cannabinoids breakdown enzyme FAAH; FAAH 385 A/A missense polymorphism is a risk factor for overweight/obesity, which are also risk factors for CAD.	[11, 45]
TRPV1	Receptor	CBD is an agonistic modulator of TRPV1; TRPV1 have a close relationship with inflammation, oxidative stress, and apoptosis.	[12–14]
5-HT1A	Receptor	CBD is an agonistic modulator of 5-HT1A; 5-HT1A mediates cell survival by activating phospholipase C/protein kinase C, calcium-calmodulin-dependent protein kinase II, and phosphatidyl inositol 3’-kinase/Akt signaling.	[13, 14]
PPARγ	Receptor	CBD is an agonistic modulator of PPARγ; The effects of CBD on anxiety, depression, the cardiovascular system, immune system, and adipogenesis are mediated, at least in part, by PPARγ.	[11, 14]
COX	Receptor	CBD affects the metabolism of arachidonic acid by affecting COX-1/-2 activity.	[11, 30]

AEA, arachidonic acid ethanolamine; CB1, cannabinoid receptor 1; CB2, cannabinoid receptor 2; COX, cyclooxygenase isoenzyme; ECS, endocannabinoid system; FAAH, fatty acid amide hydrolase; MAGL, monoacylglycerol lipase; PPARγ, peroxisome proliferator-activated receptor γ; TRPV1, transient potential vanilloid channel 1; VD, vascular disease; 2-AG, 2-acryloylglycerol; 5-HT1A, 5-hydroxytryptamine 1 A;

### CB1 and CB2 characteristics

In 1980 and 1990, CB1 and CB2 were identified and cloned separately [15, 16]. CB1 and CB2 are widely distributed, with CB1 mainly distributed in the central nervous system, and CB2 in the immune system [17, 18]. In addition, CB1 and CB2 expression in women under 50 years of age was relatively low, indicating that CB1 and CB2 expression is closely related to age and sex [19, 20]. There are many important biological processes regulated by CB1 and CB2, including nitric oxide release, activation of the mitogen-activated protein kinase (MAPK) and COX-2 pathways and protein kinase A/C, and opening of the voltage-gated Ca^2+^ and inward rectifier K^+^ channels [21, 22]. In addition, CB1 and CB2 participate in the regulation of oxidative stress and inflammation and even show opposite effects. CB1 activation promotes oxidative stress and inflammation, whereas CB2 activation mitigates oxidative stress and inflammation [23, 24]. CBD not only activates CB2 but also inhibits the activation of CB1 through allosteric regulation, thereby exerting antioxidant and anti-inflammatory effects [24, 25].

### ECS characteristics

In 2018, Di Marzo V first discovered the ECS, which mainly comprises endogenous cannabinoids, cannabinoid synthases, receptors (mainly CB1 and CB2), and metabolic enzymes. Endogenous cannabinoids include arachidonic acid ethanolamine (AEA), 2-acryloylglycerol (2-AG), oleoylethanolamide, and stearoylethanolamine. Metabolic enzymes include fatty acid amide hydrolase (FAAH) and monoacylglycerol lipase (MAGL) [26]. In humans, the ECS plays an important role in regulating oxidative phosphorylation and energy generation in mitochondria, thereby affecting the metabolism and functions of ECs and VSMCs. Given that both ECs and VSMCs play important roles in the progression of VD, it is speculated that the ECS is closely associated with VD [27, 28]. In addition, CBD does not only directly bind to CB1 and CB2 and exert a regulatory effect on the ECS, but also inhibits the activity of FAAH and reduces the decomposition of endogenous cannabinoids [11, 29, 30].

### Pharmacokinetic properties

Owing to the lack of unified regulations, multiple CBD drugs are currently being sold. These drugs can be administered orally, sublingually, via inhalation, or intravenously. Given the different CBD agents used in published studies, the reported CBD pharmacokinetics are quite different. To avoid the negative impact of combining the data of different CBD agents, we only collected and analyzed the data of Epidiolex^®^ [31–34], the only CBD drug currently approved for clinical use, to summarize the pharmacokinetic properties of CBD.

### Absorption and distribution

CBD is a lipophilic drug, 10% of which is adsorbed on the surface of erythrocytes and is rapidly distributed to tissues with rich blood supply and high-fat content, such as the brain, digestive system, and heart [8, 35]. Therefore, a high-fat diet during CBD administration can significantly increase CBD absorption.

### Bioavailability

The bioavailability of CBD is greatly affected by the route of administration; the highest bioavailability, up to 31%, was achieved via inhalation, and the peak blood concentration was reached within 3–10 min after administration. The bioavailability following oral administration was only 20%, and the time to peak blood concentration was 1–6 h, with an obvious first-pass effect [36].

### Metabolism and half-life

CBD is mainly metabolized in the liver and subsequently excreted in feces and urine. It is metabolized in the liver by cytochrome P450 (CYP450) and its isoenzymes, of which CYP3A4 and CYP2C10 are the main players [11]. The reported half-life of CBD varies greatly from study to study, ranging from 1 h to 5 days. However, some CBD metabolites (such as 6-OH-CBD, 7-OH-CBD, and 7-COOH-CBD) are biologically active and can inhibit CYP-mediated metabolism, further regulating the metabolism of CBD [11, 37].

#### Therapeutic effects of CBD on common VDs

##### Coronary artery disease (CAD)

CAD is caused by atherosclerotic plaques that lead to coronary artery stenosis or even blockage, followed by myocardial ischemia, hypoxia, and necrosis [38, 39]. Currently, sudden deaths due to CAD account for the highest number of deaths worldwide, and 12 million people are predicted to die from CAD by 2030 [40]. Research on CAD pathogenesis has yielded four etiological theories: lipid infiltration, EC injury, platelet activation, and VSMCs cloning [41–44]. Moreover, abnormal inflammation, dysfunction of lipid metabolism, and oxidative stress play important roles in the progression of CAD. CBD shows a protective effect against CAD by regulating lipid metabolism and inhibiting abnormal inflammation and oxidative stress, which were shown as follows:

### Regulation of lipid metabolism

Studies have found that genetic variations in FAAH (FAAH 385 A/A missense polymorphism) are closely related to obesity and dyslipidemia, which can increase the risk of CAD. However, genetic variation in CB2 receptor (CNR2 gene variants) does not increase the risk of CAD [45, 46]; CB2 activation can reduce CD36-dependent accumulation of oxidized low-density lipoprotein (oxLDL) and inhibit foam cell production, which in turn alleviates atherosclerotic plaque formation [47]. However, CB1 promotes atherosclerosis, and its activation can promote the production of active oxygen metabolites and accelerate cell damage and death. Studies conducted by Katsimpoulas M et al. showed that CB1 antagonists can promote the regression and stability of atherosclerotic plaque and reduce the incidence of cardiogenic stroke [48]. Since CB1, FAAH, and CB2 are all important components of ECS and have a close association with the therapeutic effect of CBD, we speculate that CBD plays a key role in regulating lipid metabolism. However, further studies are needed to confirm this speculation.

### Inhibition of abnormal inflammation

Abnormal coronary artery inflammation leads to EC dysfunction and necrosis, which, in turn, promote atherosclerotic plaque formation. Therefore, inhibiting abnormal vascular inflammation can delay the progression of CAD [42]. In vitro studies, CBD inhibited the production of pro-inflammatory mediators (such as IL-1 β, IL-6, TNF-a, and IFN- β) and upregulated the expression of JAK/STAT pathway-related anti-inflammatory mediators (such as STAT1, STAT2, SOCS3, and Cish) to alleviate inflammation induced by lipopolysaccharide [49–51]. CBD also interacts with PPARγ and/or 5-HT1A receptors to inhibit the expression of vascular adhesion molecule 1 (VCAM-1), thereby attenuating VCAM-1-mediated inflammation [52].

### Inhibition of oxidative stress

CBD has an inhibitory effect on oxidative stress, which plays an important role in the progression of CAD. In an atherosclerosis mouse model, CBD reduced the expression of superoxide dismutase, the accumulation of lipid peroxide, and the production of reactive oxygen species (ROS) [53, 54]. CBD also exerts antioxidant effects by regulating the GSH-Ps pathway [55]. The therapeutic effects of CBD and the ECS on CAD are presented in Table 2.

**Table 2:** Therapeutic effects of CBD and the ECS on vascular disease

Disease	Sample	Research Target	Summary outcome	Reference
CAD	Human; Subjects of white, black, and Asian ancestry (n = 2667);	FAAH cDNA polymorphism	FAAH 385 A/A missense polymorphism is a risk factor for overweight/obesity, which are also risk factors for CAD.	[45]
	Human; Individuals from the German MI family study (n = 1968);	CNR2 (encoding CB2) variations	Common CNR2 variations confer no susceptibility to CAD.	[46]
	Human; Overweight/obese patients (n = 6897);	Rimonabant (CB1 antagonist)	In overweight/obese patients, rimonabant induced weight loss and significant improvements in multiple cardiometabolic risk factors.	[47]
	Mouse; ApoE-/- mice (n = 48);	Exercise and rimonabant treatments	Both exercise and rimonabant treatments induced plaque regression and promoted plaque stability.	[48]
	Mouse; type I diabetic cardiomyopathy mouse model;	CBD	CBD could have a high therapeutic potential in the management of cardiovascular disorders by attenuating oxidative/nitrative stress, inflammation, cell death, and fibrosis.	[53]
	Cell; Mouse microglial cells;	CBD	CBD exerted its anti-inflammatory effects on microglia via its intrinsic antioxidant properties, which are amplified by the inhibition of glucose-dependent NADPH synthesis.	[50]
	Cell; BV-2 microglial cells;	CBD	CBD affected genes involved in the regulation of stress response and inflammation, mainly via the Nrf2/Hmox1 axis and the Nrf2/ATF4-Trib3 pathway.	[51]
Hypertension	Human; Healthy participants (n = 12);	CBD and TurboCBD™	TurboCBD™ 90 mg was associated with a slight reduction in BP.	[64]
	Human; Healthy male participants (n = 26);	CBD	CBD reduced BP at rest after a single dose, but the effect was lost after seven days of treatment.	[66]
	Human; Healthy male participants (n = 9);	CBD	Acute administration of CBD reduces resting BP and stress-induced hypertension.	[71]
	Rat; Spontaneously and deoxycorticosterone hypertensive rats;	CBD	Chronic CBD administration did not exert an antihypertensive effect in primary and deoxycorticosterone hypertension model.	[65]
	Rat; Male Wistar rats;	CBD	CBD showed anxiolytic-like properties similar to those of diazepam in a rat model of conditioned fear to context.	[70]
DRVD	Human; Noninsulin-treated type 2 diabetes patients (n = 62);	CBD	CBD decreased insulin resistance and increased glucose-dependent insulinotropic peptide levels.	[73]
	Rat; Zucker D\diabetic fatty rats	CBD	Increased circulating endocannabinoids could alter the vascular function in type 2 diabetes, possibly due to endothelium-dependent vasorelaxation improvement.	[77]
	Cell; Human coronary artery endothelial cell;	CBD	CBD could have significant therapeutic benefits against diabetic complications and atherosclerosis.	[74]
	Cell; Type I diabetic cardiomyopathy mouse model;	CBD	CBD could have a high therapeutic potential in the treatment of cardiovascular disorders, by attenuating oxidative/nitrative stress, inflammation, cell death, and fibrosis.	[53]
I/R injury	Rat; Ischemic rat hearts model;	CBD	CBD exerted a substantial in vivo cardioprotective effect.	[78]
	Rat; Male rats;	CBD	CBD showed a therapeutic effect against I/R injury-induced arrhythmias via the activation of adenosine A1 receptor.	[79]
	Rat;	CBD	CBD demonstrated a therapeutic effect against CI, possibly by diminishing TNFR1/NF-κB-induced neurotoxicity.	[81]
	Rat; Neonatal Wistar rats;	CBD	CBD administration after middle cerebral artery occlusion led to long-term functional recovery, neuronal loss and astrogliosis reduction, and modulation of apoptosis, metabolic derangement, excitotoxicity and neuro-inflammation.	[82]
	Piglet; Hypoxic-ischemic newborn piglets	CBD	The combined effect of hypothermia and CBD on excitotoxicity, inflammation, oxidative stress, and cell damage is greater than the effects of either hypothermia or CBD alone.	[83]
	Piglet; Hypoxic-ischemic newborn piglets	CBD	CBD administration after hypoxia-ischemia in piglets showed neuroprotective effects.	[84]

ApoE-/-, apolipoprotein E-deficient; Abn-CBD, abnormal cannabidiol; BP, blood pressure; CAD, coronary artery disease; CI, cerebral infarction; DRVD, diabetes-related vascular disease; ECS, endocannabinoid system; FAAH, fatty acid amide hydrolase; GPR18, G protein-coupled receptor; I/R injury, ischemia-reperfusion injury; NADPH, nicotinamide adenine dinucleotide phosphate; MI, myocardial infarction

### Hypertension

Hypertension is not only a risk factor for VD but can also lead to irreversible damage to the heart, brain, kidneys, and other organs. Dysregulation of vascular wall tension plays an important role in the pathogenesis of hypertension [56]. CBD regulates vascular wall tension via the following mechanisms: (1) In vitro, CBD directly activates CB1, G protein-coupled receptor 55 (GPR55), TRPV1, PPARs, and 5-HT1A in the vessels, causing vasodilation [57, 58]; (2) CBD activates GPR18 receptors in peripheral blood vessels and the ventrolateral central medulla oblongata, leading to vasodilation and hypotension [59, 60]; (3) CBD enhances COX activity, which increases the production of vasodilatory mediators (such as prostaglandins and nitric oxide) [61, 62]; (4) CBD regulates the function of potassium and calcium channels, thereby regulating vascular wall tension [62, 63].

Despite its ability to regulate vascular wall tension and dilate blood vessels, CBD has no or minimal effect on systolic, diastolic, or mean arterial pressure under physiological conditions. Blood pressure (BP) is regulated not only by vascular wall tension but also by cardiac contractility, heart rhythm, and the nervous system. Under physiological conditions, the body has a strong ability to regulate BP. Although CBD has a vasodilatory effect, it has a weak regulatory effect on cardiac contractility and heart rhythm under physiological conditions; therefore, CBD has no significant BP-lowering effect [64–66]. However, a study conducted by Sultan et al. showed that CBD has a favorable therapeutic effect on stress-induced hypertension [67]. Three main mechanisms account for the therapeutic effect of CBD on stress-induced hypertension: (1) CBD directly activates 5-HT1A, causing vasodilation [68]; (2) CBD has sedative properties, which can directly inhibit sympathetic nervous system activity [69]; (3) Under stressful conditions, CBD can directly reduce heart rate, cardiac output, and blood pressure [70]. In addition, other studies found that the therapeutic effect of CBD on stress-induced hypertension is affected by various factors, such as the frequency of administration (single acute administration can induce a hypotensive effect, whereas long-term administration has no obvious hypotensive effect [66]), the treatment modalities (pretreatment shows better therapeutic effect than post-treatment [66]), the route of administration (intravenous administration had the strongest hypotensive effect [68]), and the type of stress-induced hypertension (CBD has no antihypertensive effect on hypertension caused by public speeches but has an obvious antihypertensive effect on hypertension caused by sports and/or cold stimulation [71]).

Because stress-induced hypertension is a strong risk factor for acute cardiovascular events (including acute coronary syndrome and aortic dissection), we speculate that CBD could be used as an emergency medicine for the treatment of acute cardiovascular events. The therapeutic effects of CBD on hypertension are listed in Table 2.

### Diabetes-related vascular disease (DRVD)

As a strong risk factor for VD, diabetes can induce atherosclerosis, which leads to stenosis or even occlusion of the capillary artery, causing diabetic retinopathy, diabetic glomerulopathy, and diabetic foot. These pathological changes are strongly associated with EC dysfunction, vascular inflammation, and oxidative stress [72]. Therefore, blood glucose regulation is crucial in the management of DRVD. Although the administration of CBD in patients with type 2 diabetes does not reduce blood glucose levels or increase insulin sensitivity, it can alleviate vascular damage caused by diabetes [73]. Three main mechanisms account for the therapeutic effect of CBD on DRVD: (1) CBD binds to CB2 and inhibits vascular inflammation induced by hyperglycemia [53]; (2) CBD alleviates hyperglycemia-induced EC dysfunction and improves the barrier function of ECs by inhibiting hyperglycemia-induced p38 MAPK pathway activation [74, 75]; (3) CBD also inhibits oxidative stress induced by hyperglycemia (CBD works by decreasing mitochondrial superoxide production and 3-nitrotyrosine formation), thereby exerting a protective effect on ECs [76, 77]. The therapeutic effects of CBD on DRVD are shown in Table 2.

### Ischemia-reperfusion injury (I/R injury)

In clinical settings, I/R injury most commonly occurs following cerebral infarction (CI) and myocardial infarction (MI). I/R injury can further aggravate brain and heart damage via inflammation, oxidative stress, nitrative stress, and electrolyte imbalance [78]. Owing to its anti-inflammatory and antioxidant properties, CBD has a potential therapeutic role in preventing and alleviating I/R injury. The therapeutic effects of CBD on I/R injury are summarized in Table 2.

In animal models of MI, compared with post-treatment modalities, the administration of CBD before coronary artery ligation showed better therapeutic effect in narrowing the scope of MI, reducing the incidence of ventricular arrhythmias caused by MI, increasing blood flow in the perfusion-deficient area, reducing platelet aggregation, and alleviating ischemia-induced myocardial cell apoptosis [78]. The possible mechanisms of CBD on MI protection are as follows: (1) CBD directly binds to CB2 to exert anti-inflammatory effects; (2) CBD binds to the adenosine A1 receptor to inhibit the release of arrhythmogenic substances produced by platelets, thereby reducing the morbidity of ventricular arrhythmias caused by MI [79]; (3) CBD binds to 5-HT1A receptors, which dilate the capillaries in the ischemic area and increase blood flow in the perfusion-deficient area, thereby attenuating ischemia-induced myocardial cell apoptosis. In addition, the binding of CBD to 5-HT1A receptors can inhibit local platelet aggregation and thrombosis [78, 80].

Currently, most studies on the therapeutic effects of CBD in the treatment of ischemic stroke are basic research. Newborn pigs and rats are the common animals used to establish an ischemic stroke model. Studies in these models showed that pre-, post-ischemic, or pre-reperfusion administration of CBD can increase blood flow in the ischemic area, narrow the scope of ischemic stroke, maintain the function of the blood-brain barrier, and improve neurocognitive function. The protective effects of CBD on ischemic stroke are mainly achieved by binding to receptors such as CB2, 5-HT1A, and adenosine A2 [55, 81, 82]. Moreover, in the treatment of hypoxic-ischemic encephalopathy (HIE), CBD can inhibit the down-regulation of the brain’s electrical activity, alleviate neuronal metabolic damage, reduce neuronal necrosis and/or apoptosis, and inhibit inflammation and oxidative stress caused by ischemia. Therefore, the European Union has approved the use of CBD in the treatment of neonatal HIE as it can increase the therapeutic effect of hypothermia in neonatal HIE [55, 83, 84].

### Acute aortic dissection (AAD)

AAD is a critical disease characterized by rapid progression and high mortality [85]. The pathological changes in AAD mainly include vascular inflammation, apoptosis of ECs and VSMCs, and extracellular matrix degeneration. Currently, the common pharmacological therapies for AAD are sedation, analgesia, and BP control [86]. Based on the properties of CBD (such as its sedative, analgesic, anti-inflammatory, and antioxidant activities and its inhibitory effect on abnormal apoptosis of VSMCs and ECs), we speculate that CBD could serve as a potential therapeutic drug for AAD [8–10]. In addition, given that the formation and rupture of atherosclerotic plaques are strong risk factors for AAD and that CBD exerts an inhibitory effect on the formation of atherosclerotic plaques [48], we speculate that CBD can reduce AAD morbidity. Finally, as there have been no reports on the use of CBD in the treatment of AAD, we speculate that CBD could be used in the treatment of AAD based solely on its pharmacological mechanisms. Therefore, further studies are needed to determine whether CBD has a therapeutic effect on AAD.

### VD prevention

Hyperglycemia, smoking, and alcoholism are strong risk factors for VD. Studies have shown that CBD has a positive effect on these risk factors. In terms of hyperglycemia, CBD does not reduce blood glucose levels or improve insulin sensitivity; however, it inhibits vascular inflammation and ECs damage induced by hyperglycemia, which, in turn, ameliorates vascular injury caused by hyperglycemia [53, 73, 74]. With regard to smoking, a randomized, double-blind, placebo-controlled study published in 2013 showed that the inhalation of CBD reduced the prevalence of smoking by 40% among 24 participants who wished to quit smoking [87]. This result suggests that CBD facilitates active smoking cessation. In 1979, a randomized, double-blind, placebo-controlled, crossover pilot study that enrolled 10 participants with chronic alcohol abuse revealed that CBD (200 mg, orally) reduces blood alcohol concentration but does not change the effect of alcohol on behavior. This study also showed that CBD helps alleviate the damage caused by alcohol abuse [88]. As CBD showed some inhibitory effects on hyperglycemia, smoking, and alcohol abuse, we speculate that CBD may not only be useful in the treatment of VD but in its prevention.

### Side effects

In June 2018, the US Food and Drug Administration (FDA) approved Epidiolex^®^ (GW Pharmaceuticals, UK), the first CBD drug, for clinical use. Its main clinical indications include refractory epilepsy, nodular sclerosis, and Lennox–Gastaut syndrome. The most common side effects reported in the registered research on Epidiolex^®^ were diarrhea, headache, decreased appetite, and drowsiness. Based on the results of clinical trials on Epidiolex^®^, the World Health Organization in its report on CBD stated that CBD has good safety, minimal side effects, and no obvious addictive properties [36, 89].

It is worth noting that there are many non-FDA-approved CBD preparations sold worldwide. These preparations are available in various dosage forms, including oral capsules, topical creams, and tinctures, and are commonly used for cosmetic, hygienic, and nutritional support purposes [90]. Therefore, serious side effects caused by large doses of CBD are gradually being reported. For example, large doses of CBD can lead to hepatic impairment. This is because CBD is mainly metabolized in the liver and is not only a substrate of CYPs but can also simultaneously influence the secretion and function of CYP isozymes [11]. Furthermore, CBD can affect the pharmacological efficacy of some concomitant drugs, such as warfarin. CBD can enhance the antithrombotic effect of warfarin by competitively inhibiting the CYP isozymes involved in warfarin metabolism [91]. A study by Dawson in 2018 showed that administering large doses of CBD for a short period can lead to abnormal activation of CB1, which, in turn, causes stress-induced cardiomyopathy, also known as Takotsubo cardiomyopathy [92].

### Limitations

Currently, studies on the use of CBD in VD management have four limitations. First, the current evidence is only limited to the management of small vessel diseases, and reports on the use of CBD in the treatment of large vessel diseases are lacking. Second, most of the existing studies on CBD were performed on animal or cell models, and there is still a lack of rigorous controlled clinical trials to verify the therapeutic effect of CBD on VD. Third, commercially available CBD preparations differ significantly, and there is a lack of efficacy comparison between these preparations. Fourth, the pharmacological mechanism of CBD is complex; hence, more studies on the mechanism of CBD in VD treatment are needed.

### Future prospects

Although CBD has extensive regulatory effects on the cardiovascular system, most published studies have only used it in the management of cardiac diseases, and research on the management of VD with CBD remains rare. Moreover, most of the results have not been confirmed in clinical studies. To achieve the goal of using CBD in the treatment of VD, basic studies on CBD use in the management of VD, especially arterial diseases, should be conducted. Multicenter case-control studies on the role of CBD in the management of VD should also be performed.

## Conclusions

**Fig. 1:**
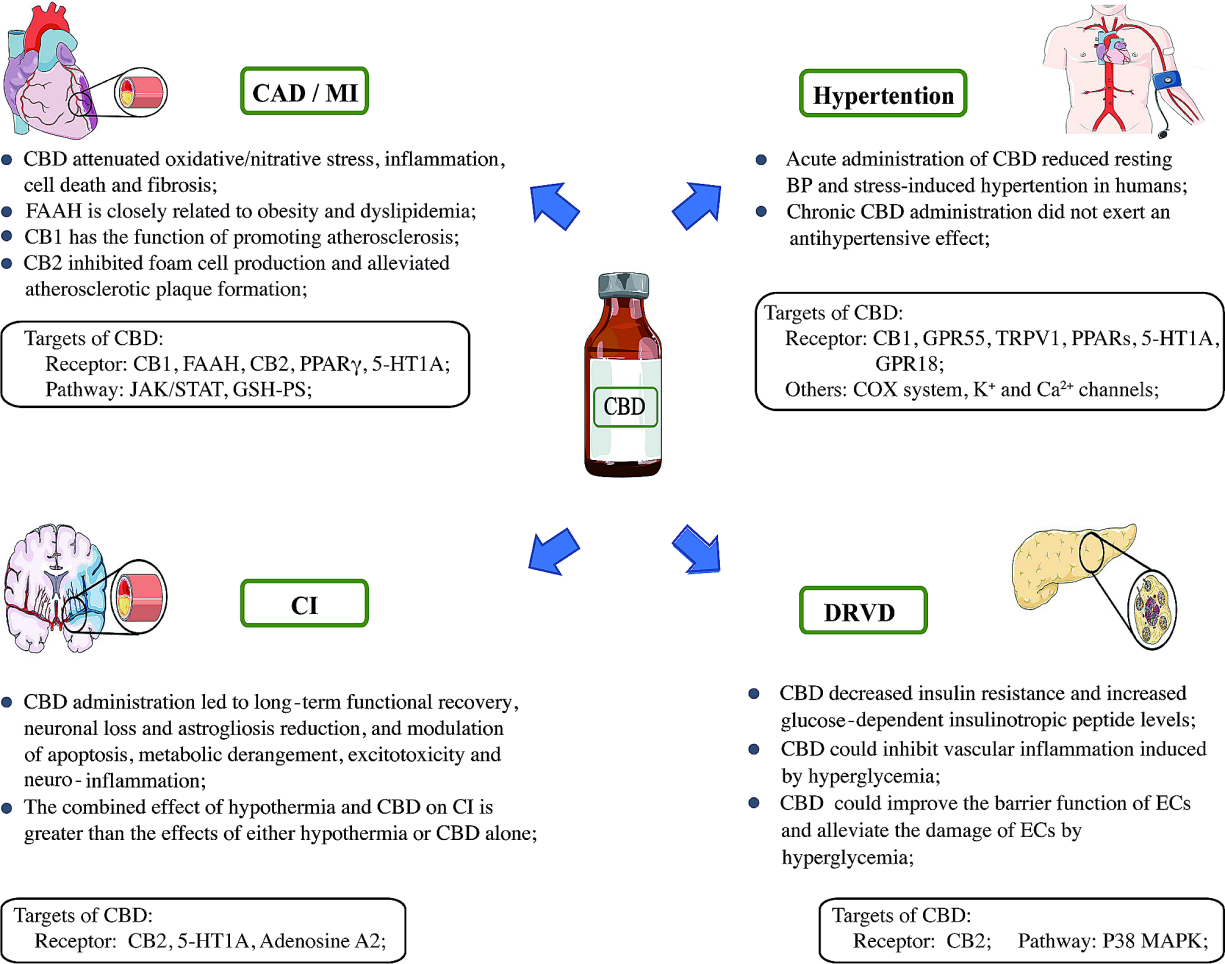
Therapeutic effects and mechanisms of CBD in common VDs. BP, blood pressure; CBD, cannabidiol; CB1, cannabinoid receptor 1; CB2, cannabinoid receptor 2; CAD, coronary artery disease; CI, cerebral infarction; DRVD, diabetes-related vascular disease; EC, endothelial cell; GPR55, G protein coupled receptor 55; GPR18, G protein coupled receptor 18; FAAH, fatty acid amide hydrolase; MI, myocardial infarction; PPAR γ, peroxisome proliferation activated receptor γ; PPAR s, peroxisome proliferation activated receptor s; TRPV1, transient potential vanilloid channel 1; VD, vascular disease; 5-HT1A, 5-hydroxytryptamine 1 A receptor;

In this review, we summarized the published data on CBD in the management of VD from four aspects: the major molecular targets of CBD in VD management, pharmacokinetic properties, therapeutic effects of CBD in common VDs, and side effects (the therapeutic effects and mechanisms of CBD in common VDs are shown in Fig. 1). According to our findings, CBD not only has therapeutic efficacy in atherosclerosis, stress-induced hypertension, DRVD, and I/R injury, but also in vascular damage caused by smoking and alcohol abuse. This review provides a theoretical foundation for further research on CBD in the management of VD.

## Data Availability

All data generated or analyzed in this study are included in this published article.

## Abbreviations

AEA: arachidonic acid ethanolamine
AAD: acute aortic dissection
BP: blood pressure
CBD: cannabidiol
CB1: cannabinoid receptor 1
CB2: cannabinoid receptor 2
CAD: coronary artery disease
CI: cerebral infarction
D9-THC: delta-9-tetrahydrocannabinol
DRVD: diabetes related vascular disease
EC: endothelial cell
ECS: endocannabinoid system
FAAH: fatty acid amide hydrolase
GPR55: G protein coupled receptor 55
HIE: hypoxic ischemic encephalopathy
I/R injury: ischemia-reperfusion injury
MAGL: monoacylglycerol lipase
MI: myocardial infarction
oxLDL: oxidized low-density lipoprotein
PPARγ: peroxisome proliferation activated receptorsγ
TRPV1: transient potential vanilloid channels 1
VSMC: vascular smooth muscle cell
VCAM-1: vascular adhesion molecule
VD: vascular disease
2-AG: 2-acryloylglycerol
5-HT1A: 5-hydroxytryptamine 1 A receptor
